# CCR6^+^ Th cells in the cerebrospinal fluid of persons with multiple sclerosis are dominated by pathogenic non-classic Th1 cells and GM-CSF-only-secreting Th cells

**DOI:** 10.1016/j.bbi.2017.03.008

**Published:** 2017-08

**Authors:** S.M. Restorick, L. Durant, S. Kalra, G. Hassan-Smith, E. Rathbone, M.R. Douglas, S.J. Curnow

**Affiliations:** a Centre for Translational Inflammation Research, Institute of Inflammation and Ageing, College of Medical and Dental Sciences, University of Birmingham, Birmingham B15 2TT, UK; b Neurology Department, University Hospital North Midlands, Stoke-on-Trent ST4 6QG, UK; c Department of Neurology, Dudley Group NHS Foundation Trust, Russells Hall Hospital, Dudley DY1 2HQ, UK; d School of Life and Health Sciences, Aston University, Birmingham B4 7ET, UK

**Keywords:** T cells, Cerebrospinal fluid, Multiple sclerosis, CCR6, Th1, Th17, GM-CSF

## Abstract

Considerable attention has been given to CCR6^+^ IL-17-secreting CD4^+^ T cells (Th17) in the pathology of a number of autoimmune diseases including multiple sclerosis (MS). However, other Th subsets also play important pathogenic roles, including those that secrete IFNγ and GM-CSF. CCR6 expression by Th17 cells allows their migration across the choroid plexus into the cerebrospinal fluid (CSF), where they are involved in the early phase of experimental autoimmune encephalomyelitis (EAE), and in MS these cells are elevated in the CSF during relapses and contain high frequencies of autoreactive cells. However, the relatively low frequency of Th17 cells suggests they cannot by themselves account for the high percentage of CCR6^+^ cells in MS CSF. Here we identify the dominant CCR6^+^ T cell subsets in both the blood and CSF as non-classic Th1 cells, including many that secrete GM-CSF, a key encephalitogenic cytokine. In addition, we show that Th cells secreting GM-CSF but not IFNγ or IL-17, a subset termed GM-CSF-only-secreting Th cells, also accumulate in the CSF. Importantly, in MS the proportion of IFNγ- and GM-CSF-secreting T cells expressing CCR6 was significantly enriched in the CSF, and was elevated in MS, suggesting these cells play a pathogenic role in this disease.

## Highlights

• CCR6^+^ T helper (Th) cells dominate the cerebrospinal fluid (CSF) in MS.
• CCR6^+^ Th17 cells are very rare.
• The dominant CCR6^+^ T cell subset secretes IFNγ and is elevated in MS CSF.
• The encephalitogenic cytokine GM-CSF is also secreted by these cells.
• GM-CSF-only secreting Th cells, also accumulate in the CSF especially in MS.

## 1 Introduction

A number of biological therapies that target the immune system have proved effective in reducing relapse rates in multiple sclerosis (MS), an inflammatory demyelinating disease of the central nervous system (CNS). However, identification and therefore specific and effective therapeutic targeting of the relevant pathogenic T cell subsets remains unresolved. Considerable attention has been given in recent years to the role of CD4^+^ T helper (Th) 17 cells in the pathogenesis of multiple sclerosis. In experimental autoimmune encephalomyelitis (EAE) disease activity is reduced in the absence of key Th17 pathway molecules including interleukin (IL) – 17A itself, IL-23 that drives the preferential accumulation of Th17 cells, and RORγt – the transcription factor required for Th17 differentiation (Rangachari and Kuchroo, 2013). Th17 cells are characterised by the production of IL-17A, as well as a number of other cytokines including IL-17F, IL-22 and IL-21, although some of these overlap with other distinct Th subsets (Annunziato et al., 2012). The chemokine receptor CCR6 is expressed by virtually all Th17 cells, along with the C-type lectin CD161 (NKR-P1A), and allows the cells to cross endothelial barriers rich in CCL20. High expression of CCL20 has been demonstrated in the choroid plexus, and in EAE CCR6 expression is required for the initial wave of T cells entering the CNS, with a later wave more dependent on CXCR3 (Liston et al., 2009, Reboldi et al., 2009), a receptor predominantly associated with Th1 cells that secrete IFNγ without IL-17A.

Multiple lines of evidence suggest a role for Th17 in MS pathogenesis, including an elevated frequency of Th17 cells in the CSF as compared to the blood, increased Th17 frequencies in both blood and CSF during relapses (Brucklacher-Waldert et al., 2009, Durelli et al., 2009), and the detection of IL-17A^+^ T cells in brain parenchyma from MS post-mortem tissue (Tzartos et al., 2008). An additional population of T cells that co-express IFNγ and IL-17A, considered to be of a Th17 lineage, can also be found in both the blood and brain in MS (Edwards et al., 2010, Kebir et al., 2009). Although there has been a strong focus on IL-17A and IFNγ production by T cells in EAE and MS, surprisingly neither cytokine is essential for the development of EAE. Instead, GM-CSF was shown to be the non-redundant cytokine required for EAE pathogenesis (Codarri et al., 2011). GM-CSF is expressed by a number of T cells subsets including Th1 and Th17, as well as a recently described population that expressed GM-CSF in the absence of both IFNγ and IL-17, termed GM-CSF-only-secreting or single-positive GM-CSF Th cells (Noster et al., 2014). Interestingly, GM-CSF secreting B cells have also now been identified in MS (Li et al., 2015). As a consequence, GM-CSF is currently a therapeutic target in a number of autoimmune and inflammatory diseases, including MS (Constantinescu et al., 2015).

Despite the prominent attention given to the role of Th17 cells in MS, much of which of has been due to their relative dominance over Th1 cells in EAE, the absolute frequencies of Th17 cells in the blood and CSF, even during MS relapses, is very low. This contrasts with the high frequency of CCR6^+^ CSF cells reported in both EAE and MS (Reboldi et al., 2009). Interestingly, CCR6^+^ myelin-reactive T cell clones derived from persons with MS produce a range of cytokines, including IFNγ, IL-17 and GM-CSF (Cao et al., 2015). Potentially pathogenic CCR6^+^ Th subsets that do not secrete IL-17A includes non-classic or non-conventional Th1 cells, a population originally characterised by the expression of CD161 and/or CCR6 (Acosta-Rodriguez et al., 2007, Becattini et al., 2015, Maggi et al., 2010, Maggi et al., 2012). Epigenetic analysis of these cells showed that they most likely originate from Th17 cells, but have lost their ability to produce IL-17A (Mazzoni et al., 2015), with studies in juvenile idiopathic arthritis demonstrating their presence in both the blood and synovial fluid. In this study we explored the association of CCR6 with the pathogenic cytokines IL-17, IFNγ and GM-CSF in CD4^+^ T cells and in particular their association with MS. We demonstrate that the dominant CSF CD4^+^ T cell subset is not composed of IL-17-secreting T cells, but non-classic/non-conventional Th1 cells as well as GM-CSF-only-secreting Th cells. These data suggest a widening of the CD4^+^ T cell subsets implicated in MS pathogenesis, with implications for their therapeutic targeting.

## 2 Methods

### 2.1 Experimental design

Ethical approval for the study was provided by the Human Biorepository Research Centre (University of Birmingham), North West Research Ethics Committee (12/NW/0828) and West Midlands Research Ethics Committee (11/WM/0206). All subjects provided written informed consent to participate in this study, in accordance with the Declaration of Helsinki. Matched peripheral blood and CSF samples were prospectively collected from persons who underwent routine diagnostic lumbar puncture, were therefore not on disease-modifying therapies at the time of sampling, and additionally had not received steroids for at least 3 months prior to sampling. Blinding was achieved as all samples were analysed before the diagnosis had been formally determined. Ethical approval for the study of peripheral blood from healthy volunteer donors was provided by the Life and Health Sciences Ethical Review Committee (University of Birmingham). Matched blood and CSF samples were collected from persons with MS (n = 13) fulfilling the diagnostic criteria according to the 2010 revisions of the McDonald criteria (Polman et al., 2011), as well as an identical number of age- and gender-matched other neurological disease controls (OND) excluding any inflammatory OND (Table 1).

**Table 1:** Characteristics of patients for peripheral blood and matched CSF analysis.

MS group			
Age*	Gender (F:M)	Disease duration (months)	EDSS at sampling
50.4 (31.1–65.5)	11:2	15 (3–276)	2.0 (0.0–6.0)

OND group			
Age*	Gender (F:M)		
47.0 (32.0–52.5)	11:2		

Diagnosis			
Recurrent intermittent, neuropathic pain, small vessel disease (4), facial pain, facial numbness, visual problems, fibromyalgia, headaches (2), idiopathic intracranial hypertension (2)			

* Median (range).

### 2.2 Preparation of CSF and peripheral blood

CSF was obtained by non-traumatic lumbar puncture, collected into polypropylene tubes, centrifuged (400*g*, 8 min), the supernatant removed and the cell pellet processed for culture and marker analysis as described below. anti-coagulated peripheral blood samples (EDTA tubes) were diluted with an equal volume of RPMI-1640 (Sigma-Aldrich), layered onto Ficoll-Paque Plus (GE Healthcare Bioscience), and centrifuged at 400*g*, 20 °C for 30 min. The peripheral blood mononuclear cell (PBMC) layer was removed and washed three times in RPMI-1640 before counting. All samples were kept at room temperature and processed within 2 h of collection. The consistency of this approach ensures that variability is kept to a minimum, and that any variation seen is equally present in the MS and control cohorts. CSF sample volumes were 5–15 ml (median 7.3 ml), yielding 400–17,000 cells (median 2750 cells).

### 2.3 Surface and intracellular marker analysis

Where indicated cells were re-suspended in RPMI-1640, 10% heat-inactivated fetal calf serum (HIFCS; Sigma-Aldrich) and stimulated with phorbol myristate acetate (PMA; 50 ng/ml), ionomycin (750 ng/ml) and Brefeldin A (2 mg/ml) (all Sigma-Aldrich) for 3 h at 37 °C, 5% CO_2_. This protocol has been widely used to reveal the cytokine production capabilities of T cells, including studies on MS (Mazzoni et al., 2015). Cells were incubated with antibodies specific for surface markers for 20 min at 4 °C before washing. To detect CCR6, cells were stained at room temperature before and after cell culture. Where required, cells were fixed and permeabilised according to the manufacturer’s instructions (FIX & PERM®, Life Technologies) before staining with antibodies specific to intracellular markers. Cells were re-suspended in phosphate-buffered saline (PBS) 2% bovine serum albumin (BSA) for flow cytometric analysis, which was performed using a CyAn™ ADP flow cytometer with data analysed using Kaluza® Flow Cytometry Analysis Software (Beckman Coulter). Isotype control antibodies or unstimulated controls (for cytokine analysis) were used to determine positivity.

### 2.4 CD4^+^ T cell positive isolation

PBMC were washed in filter-sterilized PBS, 2 mM EDTA (ethylenediamine tetra-acetic acid), 0.5% BSA (MACS buffer; 4 °C), and re-suspended in 80 μL of MACS buffer with 20 μL of CD4^+^ T Cell Microbeads (Miltenyi Biotech) per 10^7^ total cells for 20 min at 4 °C, before washing in cold MACS buffer and being re-suspended in 500 μL of MACS buffer. The cell suspension was added to a pre-rinsed MS column on a Mini-MACS separator magnet (Miltenyi Biotech). The column was washed three times with 500 μl of cold MACS buffer then removed from the magnet and the CD4^+^ fraction eluted with 1 ml MACS buffer and firm pressure on the column from a plunger. CD4^+^ T cells isolated using this method of positive selection have been shown to maintain functional activity (Akdis et al., 1995).

### 2.5 Cell migration assay

CD4^+^ T cells were positively isolated from PBMC of healthy volunteer donors as described above and re-suspended in RPMI 1640, 10% HIFCS at 2.67 × 10^6^/ml. The chemokines, CCL20 and CXCL12 (Peprotech), were diluted to the desired concentration with RPMI 1640, 10% HIFCS, and 235 μl added to the bottom well of a 5 µm 96 well HTS Transwell permeable support (Sigma-Aldrich), allowing equilibration at 37 °C, 5% CO_2_ for 1 h. 75 μl of the cell suspension was slowly added to the top well and incubated at 37 °C, 5% CO_2_ for three hours. The bottom and top compartments were re-suspended and transferred to separate tubes for stimulation with PMA and ionomycin and analysis of cytokine production as described above. AccuCheck counting beads (Thermo Fisher Scientific) were included to determine cell migration.

### 2.6 Antibodies

All antibodies were used at the concentration recommended by the manufacturer or pre-titrated for optimal staining. The following antibodies were used in this study; anti-CD3 APC-eFluor 780 (UCHT1), -CD4 APC (OKT4), -CD161 PE-Cy7 (HP-3G10), -IFNγ eFluor450 or PE (45.B3), -IL-17A eFluor 488 (eBio64DEC17), -IL-22 PE (22URT1), -IL-21 PE (eBio3A3-N2), -IL-17F PE (SHLR17), -T-bet PE (eBio4B10), -RORγt PE (AFKJS-9) (eBioscience), anti-CD3 Brilliant Violet 510 (OKT 3), -CD4 PE-Cy7 (OKT4), -CD8 Brilliant Violet 510 (RPA-T8), -CD25 PerCP-Cy5.5 (BC96), -CCR6 PE or Alexa Fluor (AF) 647 (G034E3), -CCR7 AF488 (G043H7), -CXCR3 AF647 (G025H7), anti-CD45RA PE-CF594 (HI100), -CD45RO PE-CF594 (UCHL1), -GM-CSF PE (BVD2-21C11) (BD Pharmingen), and anti-CD4 PE-Vio770 (VIT 4) (Miltenyi Biotec).

### 2.7 CD4^+^CD25^−^ T cell negative isolation

PBMC were washed in filter-sterilized PBS, 2 mM EDTA, 0.5% BSA (MACS buffer; 4 °C), and re-suspended in 90 μL of MACS buffer with 10 μL of CD4^+^ T Cell Biotin-Antibody Cocktail (Miltenyi Biotech) per 10^7^ total cells for 5 min at 4 °C. 20 μL of anti-biotin Microbeads (Miltenyi Biotech) per 10^7^ total cells was then added for 10 min at 4 °C, before increasing volume to 500 µl with MACS buffer. The cell suspension was added to a pre-rinsed LD column on a Midi-MACS separator magnet (Miltenyi Biotech). The column was washed with 1 ml of cold MACS buffer and the collected cells added to a second pre-rinsed LD column, this column was washed three times with 1 ml of cold MACS buffer to collect the unlabelled CD4^+^ fraction. CD4^+^ cells were washed and re-suspended in 90 μL of MACS buffer with 10 μL of CD25 Microbeads (Miltenyi Biotech) per 10^7^ total cells for 15 min at 4 °C, before washing in cold MACS buffer and being re-suspended in 500 μL of MACS buffer. The cell suspension was added to a pre-rinsed MS column on a Mini-MACS separator magnet (Miltenyi Biotech), and the column was washed three times with 500 µl of cold MACS buffer to collect the unlabelled CD4^+^ CD25^−^ cell fraction.

### 2.8 Purification of cytokine-secreting cells

CD4^+^CD25^−^ T cells, isolated as above, were stimulated for 3 h with PMA (50 ng/ml) and ionomycin (500 ng/ml) and cultured at a density of 5 × 10^6^ cells/cm^2^. Cells were collected from the plate by washing with cold MACS buffer (4 °C), and washed three times. Cold MACS buffer (80 μl/10^7^ cells) and each of the cytokine catch reagents (10 μl/10^7^ cells; Cytokine Secretion Assay-Cell Enrichment and Detection Kit, Miltenyi Biotech) were added and incubated at 4 °C for 5 min. RPMI 1640, 10% HIFCS warmed to 37 °C was added to dilute the cells to 10^5^/ml and incubated for 45 min at 37 °C, 5% CO_2_ spinning on a MACS Rotator (Miltenyi Biotech). Cells were kept at 4 °C for 10 min then washed in cold MACS buffer, centrifuged (300*g*, 10 min, 4 °C) and re-suspended in cold buffer at a concentration of 80 μl/10^7^ cells with 10 μl/10^7^ cells of each cytokine detection antibody. Cells were incubated at 4 °C for 10 min and washed with cold MACS buffer, before further staining as required. Cytokine positive cells were sorted using fluorescence activated cell sorting (Mo-Flo, Beckman Coulter).

### 2.9 Gene expression analysis

RNA isolation and cDNA synthesis were performed using the μMACS One-step cDNA Kit (Miltenyi Biotech) according to the manufacturer’s instructions. Synthesized cDNA was eluted from the column and collected with 50 μl cDNA Elution Buffer for quantitative real time PCR (qRT-PCR). Reactions were performed in duplex, using *GAPDH* as the control gene, in a 384 well plate with FastStart TaqMan® Probe Master Mix (Roche). All reactions were performed on a Light Cycler 480 (Roche) and analysed using the Light Cycler® 480 SW 1.5 software. The following TaqMan primer/probe sets were used (Life Technologies); *GAPDH* Hs02758991_g1 (VIC), *TBX21* Hs00203436_m1 (FAM), *RORC* Hs01076122_m1 (FAM), *IFNG* Hs00989291_m1 (FAM), *IL17A* Hs00174383_m1 (FAM). Relative gene expression (R) was analysed as 2^−[ ΔCt sample^ ^−^ ^ΔCt control]^.

### 2.10 Data analysis

Data were analysed using GraphPad Prism 6 (GraphPad Software Inc.). Statistical analysis used was as specified for each figure. The D'Agostino & Pearson omnibus normality test was used to determine if the datasets were normally distributed.

## 3 Results

### 3.1 The dominant CCR6^+^ Th subset in the CSF secretes IFNγ and is increased in MS

**Fig. 1:**
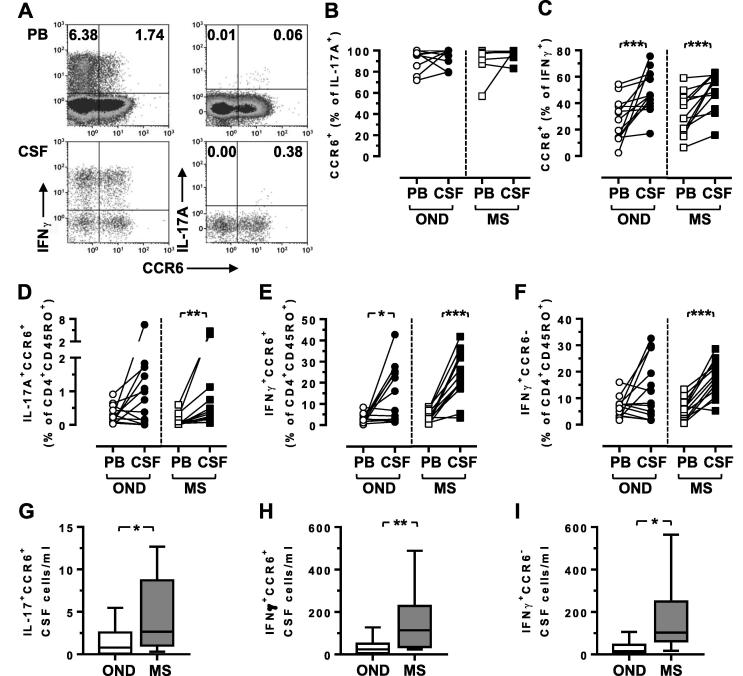
CCR6^+^ CD4^+^ Th cells in the cerebrospinal fluid predominantly secrete IFNγ, not IL-17A, and are elevated in MS. A. Representative data demonstrating CCR6 expression on IL-17^+^ and IFNγ^+^ cells (gated on CD3^+^CD45RO^+^CD8^−^ cells) in PBMC and matched CSF cells. Numbers represent the percentage of cells within the quadrant, with negative gates set based on an un-stimulated controls. B, C. The percentage of CCR6^+^ CD4^+^ T cells that expresses either IL-17A (B) or IFNγ (C) in PBMC and matched CSF. D-F. The percentage of CD4^+^ memory T cells of a CCR6^+^IL-17A^+^ (D), CCR6^+^IFNγ^+^ (E) or CCR6^−^IFNγ^+^ phenotype (F). G-I. The absolute number of CCR6^+^IL-17A^+^ (G), CCR6^+^IFNγ^+^ (H) or CCR6^−^IFNγ^+^ (I) CSF CD4^+^ memory T cells. Box and whiskers plots are shown with minimum and maximum values. Wilcoxon matched-pairs signed rank (B-F) and Mann-Whitney tests (G–I) (^*^ = p < 0.05, ^**^ = p < 0.01, ^***^ = p < 0.001); all other comparisons were non-significant (p > 0.05).

Although CCR6 is known to be expressed by a number of pathogenic and regulatory CD4^+^ Th subsets (Comerford et al., 2010), the high expression of CCR6 on CSF CD4^+^ T cells in MS has been previously attributed to IL-17-secreting Th17 cells without determination of the actual frequency of these cells (Reboldi et al., 2009). Given that IL-17-secreting CD4^+^ T cells have been reported at relatively low frequencies in the blood and CSF, even in MS (Brucklacher-Waldert et al., 2009, Durelli et al., 2009), we therefore examined the expression of both IL-17A and IFNγ in relation to the expression of CCR6. As expected all IL-17A-secreting CD4^+^ memory Th cells expressed CCR6 (Fig. 1A,B) and were present at a low frequency, consistent with previous reports in MS (Brucklacher-Waldert et al., 2009, Durelli et al., 2009). Consistent with their potential involvement in the pathogenesis of MS, the relative frequency of IL-17A^+^ CD4^+^ memory T cells in the CSF was consistently and significantly increased in MS but not OND (Fig. 1D), as well as their absolute number (Fig. 1G) as previously described (Brucklacher-Waldert et al., 2009, Durelli et al., 2009), although even in persons with MS they constituted only a small percentage of the total cells in the blood and CSF. In contrast there were much larger populations of CCR6^+^ CD4^+^ memory T cells that secreted IFNγ. The percentage of IFNγ^+^ cells that expressed CCR6 was significantly enriched within CSF as compared to the peripheral blood, although this enrichment was observed for both MS and OND cohorts (Fig. 1C); these cells represented approximately 50% of the CSF IFNγ-secreting population. CCR6^+^ IFNγ^+^ CD4^+^ memory T cells were significantly enriched in the CSF in both MS and OND, both for percentage and absolute numbers (Fig. 1E,H). Similar changes were also observed for the CCR6-IFNγ^+^ CD4^+^ memory T cells, although the increase in the OND CSF was far less consistent and not statistically significant (Fig. 1F,I).

The above data demonstrate that the previously reported increase of CCR6^+^ CD4^+^ memory T cells in the CSF (Reboldi et al., 2009) can be largely attributed to IFNγ-secreting, rather than IL-17A-secreting, T cells, and that these cells are increased in MS CSF as compared to OND. The characterisation of CCR6^+^ IFNγ CD4^+^ Th cells has been previously reported by a number of different groups, and they are referred to as non-classic Th1, ex-Th17 or non-conventional Th1 cells (Annunziato et al., 2014, Maggi et al., 2010, Maggi et al., 2012, Mazzoni et al., 2015). Consistent with the reported phenotype and potential origin of these cells we confirmed that, in our studies, cells that express CCR6 in combination with IFNγ, but which do not express IL-17A, expressed a combination of transcription factors that are associated with Th1 and Th17 subsets (Fig. S1).

### 3.2 CCR6 expressed on IFNγ-secreting CD4^+^ memory T cells is functional

**Fig. 2:**
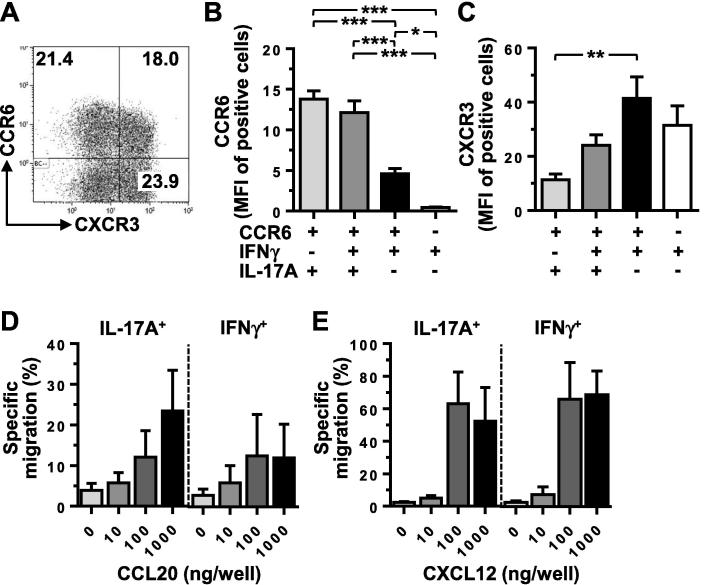
CCR6 expressed on IFNγ^+^IL-17A^−^ CD4^+^ memory T cells is functional. A. Expression of CXCR3 and CCR6 gated on CD4^+^CD45RO^+^ memory T cells. B, C. Median fluorescence intensity (MFI) of CCR6 (B) and CXCR3 (C) in CD4^+^CD45RO^+^ memory T cells expressing combinations of CCR6, IFNγ and IL-17A. Repeated measures ANOVA with Bonferroni's multiple comparison test (n = 5); ^*^ = p < 0.05, ^**^ = p < 0.01, ^***^ = p < 0.001. All other comparisons were non-significant (p > 0.05). D, E. Purified CD4^+^ T cells were migrated in a transwell in response to the indicated concentrations of CCL20 (D) or CXCL12 (E). The migrated and non-migrated fractions were then stimulated with PMA and ionomycin before determining IFNγ and IL-17 expression, allowing calculation of the migration of each subset. Data represent the mean and SEM of three independent experiments.

Although CCR6 was clearly expressed on a subset of IFNγ-secreting CD4^+^ memory T cells, consistent with a number of published studies, it has yet to be established whether the receptor is functional, especially given the significantly reduced intensity of CCR6 expression observed on these cells (Fig. 2A-B). Migration assays using purified CD4^+^ T cells demonstrated that both IL-17A^+^IFNγ^−^ and IFNγ^+^IL-17A^−^ T cells migrated towards CCL20 in a dose-dependent manner, as well as responding to the control chemokine CXCL12 which acts through CXCR4 found uniformly on all subsets (Fig. 2D-E). The response to CCL20 for the IFNγ-secreting T cells appeared to be lower than for the Th17 cells (Fig. 2D), consistent with the observed reduction in median fluorescence intensity for CCR6, whereas there was no difference in their ability to migrate in response to CXCL12 (Fig. 2E).

### 3.3 GM-CSF-secreting Th cells are elevated in MS

Collectively, our data suggest that CCR6^+^ IFNγ-secreting CD4^+^ T cells have both the chemokine receptor expression and cytokine secretion capabilities that would allow them to both enter the CNS, at least at the level of the choroid plexus, and mediate either immune surveillance functions, or if autoreactive, a pathogenic role.

The presence of CCR6 on a subset of IFNγ-secreting CD4^+^ T cells, with absent IL-17A production, indicates that this subset shares features of both Th17 and Th1 cells, as reported in a number of studies (Maggi et al., 2010, Maggi et al., 2012). We confirmed the absence of a number of Th17-associated cytokines, IL-17F, IL-21 and IL-22 within the CCR6^+^ IFNγ^+^ subset, despite them being produced by both IL-17^+^ and IL-17^+^IFNγ^+^ CCR6^+^ subsets (Fig. S2A). As part of this analysis we also examined the expression of GM-CSF, and confirmed its broad expression (Fig. S2B-C).

**Fig. 3:**
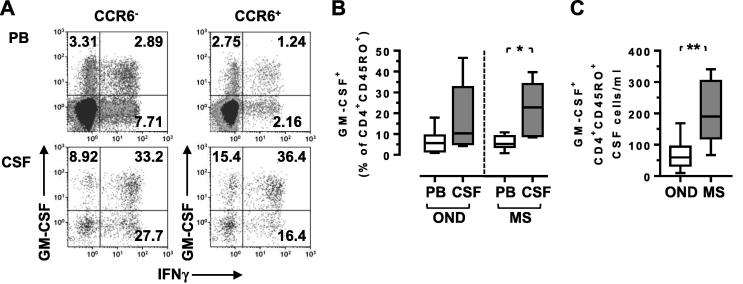
CCR6^+^IFNγ^+^ CD4^+^ memory T cells in the CSF secrete GM-CSF and are elevated in MS. Matched PBMC and CSF cells were stimulated with PMA and ionomycin and stained for surface CCR6, and intracellular IFNγ, IL-17 and GM-CSF. Representative data is shown from a person with MS (A). All samples were gated on CD3^+^CD4^+^CD45RO^+^ memory T cells. Box and whiskers plots are shown with minimum and maximum values. Wilcoxon matched-pairs signed rank test (B,C); ^*^ = p < 0.05, ^**^ = p < 0.01. All other comparisons were non-significant (p > 0.05).

Because GM-CSF is thought to be a key encephalitogenic cytokine, being non-redundant in EAE, we evaluated its expression in both IL-17A- and IFNγ-secreting cells, as well as those cells that secrete neither of these cytokines, previously referred to as GM-CSF-only-secreting or GM-CSF single positive Th cells (Herndler-Brandstetter and Flavell, 2014, Noster et al., 2014, Sheng et al., 2014). In a cohort of persons with MS, the frequency of GM-CSF^+^ CD4^+^ memory T cells was significantly increased in the CSF as compared to the blood, with a lower and non-significant increase in the OND cohort (Fig. 3A,B). Although these data are consistent with previously reported data (Noster et al., 2014), we failed to demonstrate a significant difference between the CSF of MS and OND cohorts when measuring the frequency of these cells (p = 0.079), although there was a significant increase in their numbers (Fig. 3C). There was also clear evidence that some individuals in the OND cohort had high frequencies of GM-CSF^+^ CD4^+^ memory T cells in their CSF (Fig. 3B).

**Fig. 4:**
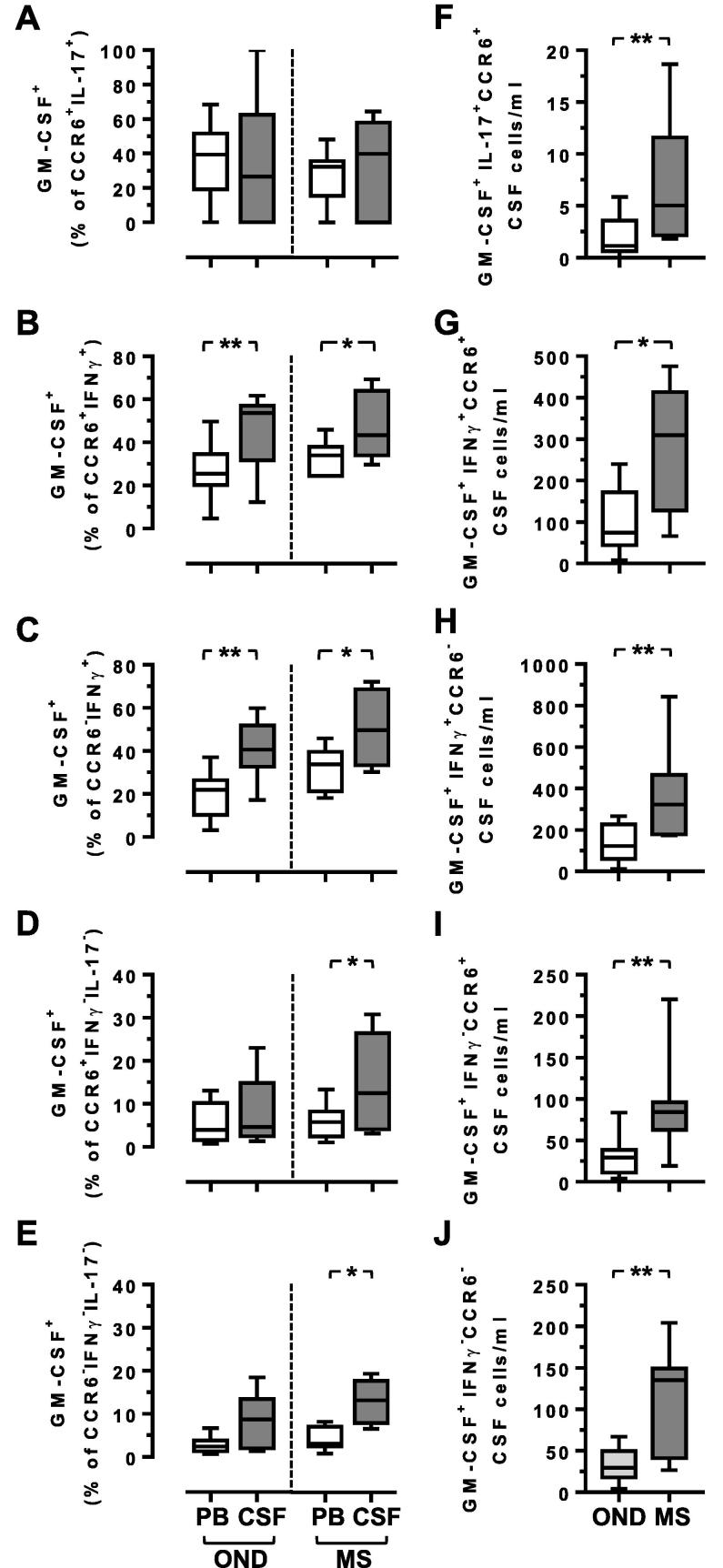
CCR6^+^ CD4^+^ GM-CSF-only-secreting T cells are elevated in MS. The percentage (A–E) of CD4^+^ memory T cells expressing the indicated combinations of CCR6, IL-17A, IFNγ and GM-CSF in PBMC and matched CSF. The absolute numbers of CSF CD4^+^ memory T cells of each phenotype are shown in F-J. Box and whisker plots are shown with minimum and maximum values for each cohort. Wilcoxon matched-pairs signed rank (A-E) and Mann-Whitney tests (B–J) (^*^ = p < 0.05, ^**^ = p < 0.01); all other comparisons were non-significant (p > 0.05).

We next determined which Th subsets were contributing to the elevations in GM-CSF^+^ CD4^+^ memory T cells in the CSF of persons with MS. CCR6^+^IL-17^+^ CD4^+^ T cells in both OND and MS cohorts, and in both the blood and CSF, were capable of secreting GM-CSF to similar degrees (Fig. 4A). By contrast the frequencies of CCR6^+^IFNγ^+^ cells that can produce GM-CSF were significantly elevated in the CSF, as compared to the blood, although this was equally elevated in the OND and MS cohorts (Fig. 4B). A similar pattern was also observed for CCR6^−^IFNγ^+^ CD4^+^ T cells (Fig. 4C). When considering the numbers of these cells, there were significant increases for all GM-CSF-secreting populations of CSF CD4^+^ T cells in MS, as compared to OND (Fig. 4F–H). However, differences were observed when considering the IL-17^−^IFNγ^−^ CD4^+^ T cells that express GM-CSF. This population has previously been identified and referred to as GM-CSF-only-secreting (Herndler-Brandstetter and Flavell, 2014). There was a significant increase in the CSF of persons with MS for the CCR6^+^IL-17^−^IFNγ^−^GM-CSF^+^ CD4^+^ T cell subset, both for percentage and frequency (Fig. 4D), as well as for the equivalent CCR6^−^ population (Fig. 4E), although again elevated in absolute numbers in both cohorts (Fig. 4I-J). These data demonstrate that there are increases in GM-CSF secreting T cells in the CSF of persons with MS, but that this increase is largely confined to CD4^+^ T cells that do not express either IFNγ or IL-17.

Overall, these data suggest that there is a selective recruitment of CCR6^+^ IFNγ-secreting CD4^+^ memory T cells to the CSF that is increased in MS, that these cells are able to produce the encephalitogenic cytokine GM-CSF, and that these cells constitute the dominant CCR6^+^ CD4^+^ Th subset in the CSF in MS.

## 4 Discussion

There is a large body of literature implicating CD4^+^ Th17 cells in the pathogenesis of MS (Brucklacher-Waldert et al., 2009, Cao et al., 2015, Durelli et al., 2009, Kebir et al., 2009, Li et al., 2011, Tzartos et al., 2008). These data include the role of these cells in EAE, where they have been shown to enhance disease by driving the first phase of recruitment across the choroid plexus barrier (Liston et al., 2009, Reboldi et al., 2009). In MS, Th17 cells are increased in the CSF and peripheral blood during relapses (Brucklacher-Waldert et al., 2009, Durelli et al., 2009), and are present in in parenchymal post-mortem tissue (Tzartos et al., 2008). CCR6 is a well validated marker expressed on virtually all Th17 cells, and myelin/MOG-specific T cells are found within the CCR6^+^ subset (Cao et al., 2015, Sallusto et al., 2012). Consequently, some studies have relied on the expression of CCR6 to indicate the presence of Th17 cells. In particular the frequency of CCR6^+^ T cells in MS CSF was suggested to indicate the dominance of Th17 cells (Reboldi et al., 2009). However, it is now clear that CCR6 is not restricted to Th17 cells, but can include both regulatory cells and other potentially pathogenic subsets including IFNγ- and GM-CSF-secreting CD4^+^ T cells. In this study we have determined that the dominant CCR6^+^ CD4^+^ T cell subset in MS is not the Th17 subset but either non-classic Th1 cells that secrete IFNγ and GM-CSF, or GM-CSF-only-secreting CD4^+^ Th cells.

A number of studies have characterised a population of Th1 cells termed non-classic or non-conventional, that express many markers associated with Th17 cells, but importantly do not produce IL-17A (Annunziato et al., 2014, Maggi et al., 2014, Maggi et al., 2010, Maggi et al., 2012), and our own data were consistent with the published literature (Figs. 1, S1,S2). These cells are likely to have been derived from Th17 cells, as the IL-17A locus remains partially de-methylated and they share many transcriptional features of Th17 cells (Annunziato et al., 2014, Becattini et al., 2015, Mazzoni et al., 2015). The origin of these cells is consistent with studies in mice where fate-mapping of Th17 cells revealed their ability to differentiate to a Th1 phenotype, and interestingly this was particular prevalent in EAE (Hirota et al., 2011). The presence of high numbers of CCR6^+^ Th1 cells in the CSF reveals their ability to efficiently migrate across the choroid plexus. The ligand for CCR6, CCL20, has been previously found at high levels in the choroid plexus suggesting that CCR6 itself is involved in their migration (Reboldi et al., 2009). Although we observed a reduced expression of CCR6 on IFNγ-secreting as compared to IL-17A-secreting CD4^+^ memory T cells, we were able to demonstrate that this receptor is functional, suggesting that CCR6 may participate in their migration across through the choroid plexus. It is likely that CXCR3 also participates in this migration process, as non-classic Th1 cells also express CXCR3 (Maggi et al., 2012) and virtually all cells arriving in the CSF are CXCR3^+^ (Kivisakk et al., 2002).

The two-step model proposed based on data from EAE models suggests that Th17, or at least CCR6^+^ cells, are required for the first phase of disease being recruited across the choroid plexus and/or meninges (Reboldi et al., 2009). Once a few pathogenic T cells have migrated through into the CNS and been re-activated by antigen this would then drive activation of the parenchymal endothelial barrier, reducing barrier integrity, and allowing a larger influx of Th1 cells to enter during a second phase of disease; this scenario might therefore suggest that CCR6^+^ Th1 cells are involved early in the disease process. An alternative hypothesis can be suggested based on data using a human TCR transgenic mouse that develops spontaneous disease. In this model Th1 cells are involved earlier in disease and then Th17 cells at a later stage when the tissue becomes significantly affected (Lowther et al., 2013). We do not have longitudinal data available in MS, but a much larger study comparing different stages of MS may shed some light on when non-classic Th1 cells are acting. It will also be of interest to determine if there is any prognostic relevance to their frequencies within the CSF. Although we have demonstrated a high frequency of CCR6^+^ Th1 cells in the CSF of persons with MS, there were also substantial numbers in a range of other non-inflammatory neurological diseases. It is therefore likely that these cells are also recruited in the absence of inflammation to provide an immune surveillance role, with frequencies then increasing during episodes of inflammation. Although none of the MS or control cohort were on any immunomodulatory treatment at the time of sampling or during the preceding 3 months, we cannot exclude a possible influence of any historical treatments. It would be interesting in future studies, particularly in MS, to examine the impact of distinct immunomodulatory treatments.

Although both IFNγ-secreting Th1 and IL-17A-secreting Th17 cells infiltrate the CNS in MS and EAE, neither cytokine is absolutely required for the induction of EAE. The ultimate pathogenicity of CD4^+^ T cells is thought instead to relate to the production of GM-CSF (Codarri et al., 2011). GM-CSF can be associated with either Th1 or Th17 cells, including non-classic Th1 cells (Piper et al., 2014), as well as a more recently described population of STAT5 regulated GM-CSF-only-producing Th cells (Herndler-Brandstetter and Flavell, 2014, Noster et al., 2014, Piper et al., 2014, Sheng et al., 2014). Elevations of GM-CSF secreting T cells were previously observed in MS CSF (Noster et al., 2014), but this was not attributed to a distinct Th subset. Our data demonstrate that the dominant GM-CSF-secreting Th subsets are non-classic Th1 cells and GM-CSF-only-secreting Th cells, being elevated in the CSF as compared to the blood, and with elevated levels in MS as compared to disease controls. There are of course some limitations to our study. Further studies would be required to determine the influence of disease duration, treatment regimens and impact of relapse, in larger cohorts. Additionally, the analysis of CSF, although clearly reflecting activity within CNS tissues may not fully parallel populations observed in the tissue.

## 5 Conclusions

Our data have important implications not only for our understanding of the pathogenesis of MS, but also for therapeutic strategies. Dramatic reductions in relapse rates have been achieved by preventing T cell infiltration into the CNS, and there is emerging evidence that targeting IL-17A might have some efficacy in MS (Havrdova et al., 2016). The close transcriptional associations between Th17 and non-classic Th1 cells might suggest that approaches targeting shared pathways in these cells might also be of benefit. For example, it is possible that RORγt antagonists may not only impact Th17 cells (Isono et al., 2014) but also the non-classic Th1 lineage, and thereby impact on the pathogenic autoreactive T cells contained within the CCR6^+^ T cell subset (both Th17 and non-classic Th1). Even if RORγt antagonists only impact on Th17 cells, with chronic therapy, over time there may be an impact on the conversion of Th17 cells to non-classic Th1 cells. Further investigations will be required to determine the relative impact of all Th17 pathway modulators on Th17 and CCR6^+^ Th1 cells, as well as the potential of targeting GM-CSF (Wicks and Roberts, 2016).
