# Supplementary material for: CCR6+ Th cells in the cerebrospinal fluid of persons with multiple sclerosis are dominated by pathogenic non-classic Th1 cells and GM-CSF-only-secreting Th cells
Source: Brain Behav Immun. 2017 Aug;64:71–9. doi: 10.1016/j.bbi.2017.03.008 (PMC5490506; doi:10.1016/j.bbi.2017.03.008)
Supplement: Supplementary data 1 [file mmc1.pptx]

## Slide 1
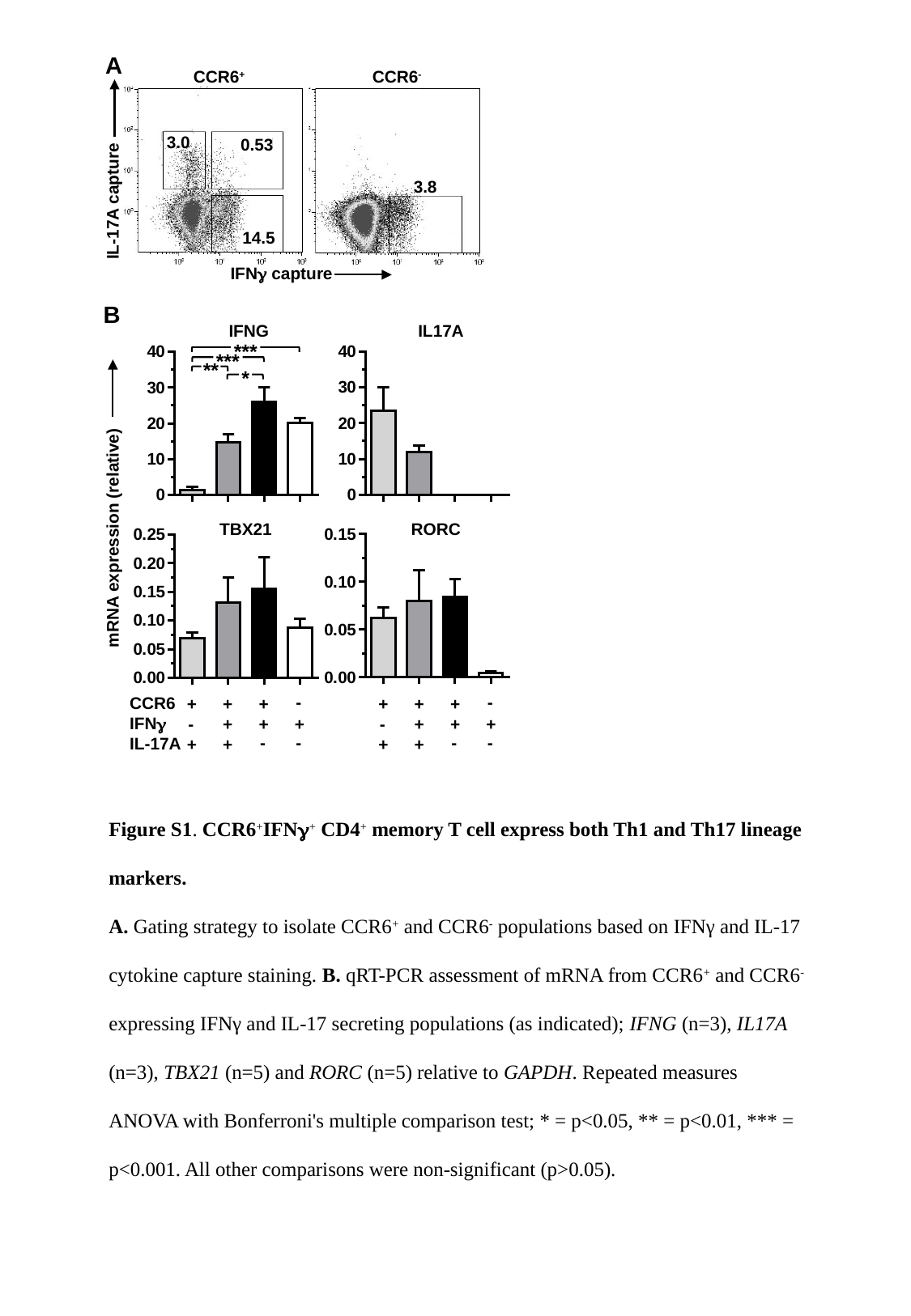

A
CCR6+
CCR6-
IL-17A capture
3.0
0.53
14.5
3.8
IFNg capture
B
IFNG
IL17A
***
***
**
*
mRNA expression (relative)
TBX21
RORC
-
+
-
+
-
+
+
+
+
+
+
-
-
+
-
+
-
+
+
+
+
+
+
-
CCR6
IFNg
IL-17A
Figure S1. CCR6+IFNg+ CD4+ memory T cell express both Th1 and Th17 lineage markers.
A. Gating strategy to isolate CCR6+ and CCR6- populations based on IFNγ and IL-17 cytokine capture staining. B. qRT-PCR assessment of mRNA from CCR6+ and CCR6- expressing IFNγ and IL-17 secreting populations (as indicated); IFNG (n=3), IL17A (n=3), TBX21 (n=5) and RORC (n=5) relative to GAPDH. Repeated measures ANOVA with Bonferroni's multiple comparison test; * = p<0.05, ** = p<0.01, *** = p<0.001. All other comparisons were non-significant (p>0.05).

## Slide 2
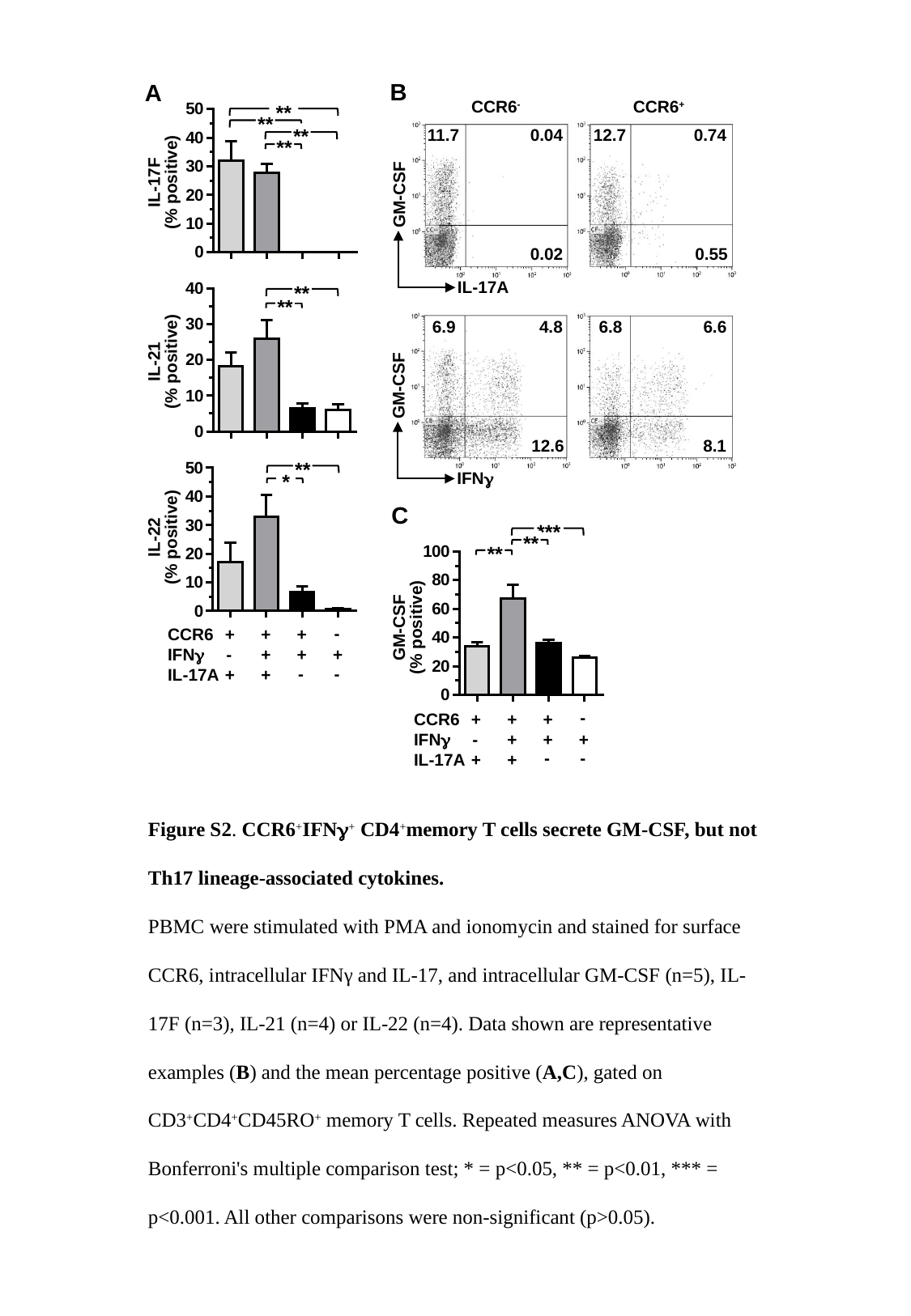

B
CCR6-
CCR6+
12.7
0.74
0.55
11.7
0.04
0.02
GM-CSF
IL-17A
6.9
4.8
6.8
6.6
GM-CSF
IFNg
12.6
8.1
A
**
**
**
**
IL-17F
(% positive)
**
**
IL-21
(% positive)
**
*
C
***
IL-22
(% positive)
**
**
GM-CSF
(% positive)
-
+
-
+
-
+
+
+
+
+
+
-
CCR6
IFNg
IL-17A
-
+
-
+
-
+
+
+
+
+
+
-
CCR6
IFNg
IL-17A
Figure S2. CCR6+IFNg+ CD4+memory T cells secrete GM-CSF, but not Th17 lineage-associated cytokines.
PBMC were stimulated with PMA and ionomycin and stained for surface CCR6, intracellular IFNγ and IL-17, and intracellular GM-CSF (n=5), IL-17F (n=3), IL-21 (n=4) or IL-22 (n=4). Data shown are representative examples (B) and the mean percentage positive (A,C), gated on CD3+CD4+CD45RO+ memory T cells. Repeated measures ANOVA with Bonferroni's multiple comparison test; * = p<0.05, ** = p<0.01, *** = p<0.001. All other comparisons were non-significant (p>0.05).
